# Supplementary material for: Association of Gastric Myoelectric Activity with Dietary Intakes, Substrate Utilization, and Energy Expenditure in Adults with Obesity
Source: Nutrients. 2022 Sep 28;14(19):4021. doi: 10.3390/nu14194021 (PMC9572295; doi:10.3390/nu14194021)
Supplement: Supplementary file 1 [file nutrients-14-04021-s001.zip › nutrients-1832948-supplementary.pdf]

Supplementary Materials

Table S1. Dietary intake differences stratified by subgroups, n=115.

Variables	HW (n=24) Mean ± SD	OW (n=29) Mean ± SD	OB (n=41) Mean ± SD	MO (n=21) Mean ± SD	*P*-value
Kcal	1389.13 ± 362.53 ^{a}	1435.99 ± 454.84 ^{a}	1959.71 ± 511.20 ^{b}	2042.26 ± 772.12 ^{b}	< 0.001 **
Kcal per fat	474.13 ± 157.06 ^{a}	449.26 ± 202.57 ^{a}	620.05 ± 212.03 ^{b}	619.67 ± 250.50 ^{b}	0.001 *
Kcal per Sat. fat	148.46 ± 69.14 ^{a}	129.13 ± 68.49 ^{a}	199.67 ± 87.83 ^{b}	204.74 ± 76.09 ^{b}	< 0.001 **
Protein (g)	67.74 ± 37.30 ^{a}	64.74 ± 32.72 ^{a}	95.38 ± 39.75 ^{b}	90.23 ± 44.88 ^{b}	0.003 *
CHO (g)	156.44 ± 50.19 ^{a}	178.74 ± 57.08 ^{a}	228.25 ± 65.28 ^{b}	242.50 ± 108.10 ^{b}	< 0.001 **
Fiber (g)	8.20 ± 4.52 ^{a}	11.90 ± 4.51 ^{b}	13.05 ± 6.69 ^{b}	11.25 ± 6.50 $^{a\ b}$	0.014 *
Soluble Fiber (g)	0.29 ± 0.27 ^{a}	0.39 ± 0.64 ^{a}	0.31 ± 0.49 ^{a}	0.40 ± 0.41 ^{a}	0.797
Total sugars	56.25 ± 27.56 ^{a}	54.28 ± 21.88 ^{a}	60.95 ± 23.13 ^{a}	76.41 ± 40.38 ^{b}	0.04 *
Added sugars	17.76 ± 18.61 ^{a}	15.57 ± 13.05 ^{a}	18.63 ± 20.91 ^{a}	35.43 ± 30.03 ^{b}	0.006 *
Monosaccharide	2.47 ± 4.46 ^{a}	1.70 ± 2.96 ^{a}	2.60 ± 4.14 ^{a}	6.27 ± 12.53 ^{b}	0.08
Disaccharide	2.35 ± 3.57 $^{a\ b}$	2.27 ± 4.51 $^{a\ b}$	2.09 ± 2.59 ^{a}	4.15 ± 4.37 ^{b}	0.20
Other CHO	72.59 ± 34.46 ^{a}	92.84 ± 53.82 $^{a\ b}$	114.07 ± 56.42 ^{b}	123.84 ± 82.43 ^{b}	0.01 *
Fat (g)	52.70 ± 17.48 ^{a}	49.92 ± 22.51 ^{a}	68.90 ± 23.57 ^{b}	68.85 ± 27.83 ^{b}	0.001 **
Sat. Fat (g)	16.50 ± 7.68 ^{a}	14.35 ± 7.61 ^{a}	22.18 ± 9.76 ^{b}	22.75 ± 8.45 ^{b}	< 0.001 **
MUF	9.45 ± 5.14 $^{a\ c}$	9.06 ± 5.54 ^{a}	12.80 ± 6.45 ^{b}	12.84 ± 7.12 $^{b\ c}$	0.02 *
PUF	5.99 ± 4.68 ^{a}	7.23 ± 5.18 $^{a\ b}$	8.57 ± 4.99 ^{b}	9.20 ± 5.84 ^{b}	0.13
Trans fat	0.23 ± 0.30 $^{a\ b}$	0.13 ± 0.15 ^{a}	0.22 ± 0.27 $^{a\ b}$	0.30 ± 0.35 ^{b}	0.16
Cholesterol	331.92 ± 252.84 ^{a}	223.55 ± 196.37 ^{b}	323.68 ± 161.63 ^{a}	285.07 ± 172.99 $^{a\ b}$	0.13
Water foods	474.49 ± 274.94 ^{a}	469.80 ± 245.35 ^{a}	528.21 ± 257.05 ^{a}	723.03 ± 410.45 ^{b}	0.01 *
Fat-soluble vitamin					
Vitamin A	1626.34 ± 3143.29 ^{a}	1677.70 ± 2831.73 ^{a}	1151.48 ± 1060.28 ^{a}	1600.75 ± 2198.68 ^{a}	0.76
Carotenoid	51.13 ± 114.54 ^{a}	77.13 ± 126.23 ^{a}	62.18 ± 101.32 ^{a}	90.77 ± 164.40 ^{a}	0.72
Retinol	213.46 ± 542.26 ^{a}	137.89 ± 274.98 ^{a}	285.86 ± 611.37 ^{a}	309.40 ± 629.87 ^{a}	0.62
B-carotene	894.03 ± 1828.91 ^{a}	755.96 ± 902.88 ^{a}	475.67 ± 988.47 ^{a}	703.41 ± 894.60 ^{a}	0.55
Vitamin D	40.18 ± 55.80 ^{a}	22.05 ± 54.56 ^{a}	18.80 ± 25.00 ^{a}	38.79 ± 59.62 ^{a}	0.21
Vitamin E	2.84 ± 1.69 ^{a}	3.03 ± 2.08 ^{a}	3.51 ± 3.14 ^{a}	3.33 ± 3.05 ^{a}	0.76
Vitamin K	51.87 ± 60.40 ^{a}	38.68 ± 48.67 ^{a}	36.47 ± 45.28 ^{a}	38.52 ± 27.52 ^{a}	0.62
Water-soluble vitamin					
Vitamin C	39.35 ± 51.94 ^{b}	67.41 ± 66.90 ^{a}	32.87 ± 37.45 ^{b}	29.34 ± 25.41 ^{b}	0.01 *
Vitamin B1	4.08 ± 16.63 ^{a}	0.76 ± 0.30 ^{a}	0.97 ± 0.53 ^{a}	1.16 ± 0.80 ^{a}	0.36
Vitamin B2	2.13 ± 6.73 ^{a}	0.89 ± 0.44 ^{a}	1.08 ± 0.52 ^{a}	1.16 ± 0.68 ^{a}	0.49
Vitamin B3	19.26 ± 17.26 $^{a\ b}$	17.15 ± 7.59 ^{a}	25.08 ± 15.08 ^{b}	24.64 ± 15.21 $^{a\ b}$	0.08
Vitamin B6	1.88 ± 3.81 ^{a}	0.93 ± 0.67 ^{a}	1.19 ± 1.03 ^{a}	1.27 ± 0.70 ^{a}	0.35
Vitamin B12	3.22 ± 5.73 ^{a}	1.90 ± 3.25 ^{a}	3.01 ± 6.73 ^{a}	3.89 ± 5.63 ^{a}	0.66
Vitamin biotin	2.38 ± 2.16 $^{a\ b}$	2.85 ± 1.85 ^{a}	1.39 ± 1.10 ^{b}	3.21 ± 3.56 ^{a}	0.04
Folate	103.90 ± 76.00 ^{a}	151.92 ± 91.76 $^{a\ b}$	166.30 ± 125.05 ^{b}	196.25± 199.15 ^{b}	0.10
Vitamin B5	1.50 ± 1.63 ^{a}	1.38 ± 1.22 ^{a}	2.18 ± 1.88 ^{a}	2.00 ± 1.93 ^{a}	0.23
Other micronutrients					
Omega-3	2.82 ± 9.74 ^{a}	0.63 ± 0.57 ^{a}	1.06 ± 1.57 ^{a}	0.75 ± 0.47 ^{a}	0.30
Omega-6	6.65 ± 6.89 ^{a}	6.22 ± 4.69 ^{a}	7.90 ± 5.07 ^{a}	8.07 ± 5.40 ^{a}	0.51
Caffeine	55.66 ± 64.78 ^{a}	63.81 ± 85.99 ^{a}	53.41 ± 58.70 ^{a}	63.41 ± 53.12 ^{a}	0.90
Choline	124.30 ± 133.49 ^{a}	113.78 ± 85.71 ^{a}	159.96 ± 129.46 ^{a}	169.77 ± 132.39 ^{a}	0.27
Ca	480.74 ± 277.81 $^{a\ b}$	390.15 ± 231.36 ^{a}	573.25 ± 297.76 ^{b}	538.68 ± 344.35 $^{a\ b}$	0.07
Chromium	1.46 ± 0.87 ^{a}	1.39 ± 0.94 ^{a}	1.44 ± 0.90 ^{a}	1.89 ± 0.99 ^{a}	0.50
Copper	0.58 ± 1.02 ^{a}	0.68 ± 0.57 ^{a}	0.90 ± 1.23 ^{a}	0.95 ± 1.11 ^{a}	0.54
Fluoride	0.006 ± 0.005 ^{a}	0.001 ± 0.003 ^{a}	0.011 ± 0.019 $^{a\ b}$	0.023 ± 0.038 ^{b}	0.25
Iodine	8.23 ± 10.33 ^{a}	7.42 ±11.08 ^{a}	3.98 ± 3.05 ^{a}	7.54 ± 4.25 ^{a}	0.45
Iron	6.55 ± 2.71 ^{a}	7.24 ± 2.82 ^{a}	9.55 ± 4.82 ^{b}	11.09 ± 6.66 ^{b}	0.002 *
Mg	104.89 ± 65.25 ^{a}	127.70 ± 54.99 $^{a\ b}$	143.56 ± 66.68 ^{b}	146.07 ± 72.03 ^{b}	0.09
Manganese	0.60 ± 0.44 ^{a}	0.75 ± 0.47 ^{a}	0.90 ± 0.76 ^{a}	0.92 ± 0.75 ^{a}	0.28
Molybdenum	0.58 ± 0.40 ^{a}	3.06 ± 3.28 ^{b}	0.38 ± 0.37 ^{a}	1.44 ± 1.55 $^{a\ b}$	0.005 *
Phosphorus	496.65 ± 323.25 ^{a}	1462.45 ± 2128.50 ^{b}	819.88 ± 348.61 ^{a}	820.50 ± 470.11 ^{a}	0.02

K	1145.53 ± 607.56 [a]	1321.13 ± 576.02 [a]	1590.91 ± 801.26 [b]	1795.71 ± 680.43 [b]	0.007*
Selenium	59.14 ± 48.39 [a]	69.708 ± 29.56 [a]	92.09 ± 47.35 [b]	84.91 ± 54.60 [a b]	0.03
Na	2002.16 ± 905.53 [a]	2415.18 ± 1137.40 [a c]	3314.7 ± 1727.20 [b]	3044.77 ± 2144.46 [b c]	0.006*
Zinc	3.45 ± 2.54 [a]	3.91 ± 1.68 [a]	5.50 ± 3.07 [a]	17.97 ± 49.13 [b]	0.07

Different superscripts (a, b, and c) indicate statistically different. Ca, calcium; CHO, carbohydrate; K, potassium; Kcal, calorie; Mg, magnesium; MUF, monounsaturated fat; Na, sodium; PUF, polyunsaturated fat Sat. fat, saturated fat* *$P < 0.05$ (2-tailed); ** $P < 0.001$ (2-tailed)*
